# The Roles of USG and NCCT in the Diagnosis of Acute Appendicitis: A Study in a Tertiary Care Center in North Eastern India

**DOI:** 10.4314/ejhs.v33i4.14

**Published:** 2023-07

**Authors:** Uddalok Das, Amarendra Nath Sarkar, Dilip Chandra Barman, Narayan Pandit

**Affiliations:** 1 Department of Radiodiagnosis, North Bengal Medical College and Hospital; 2 Department of General Surgery, North Bengal Medical College and Hospital; 3 Department of Pathology, North Bengal Medical College and Hospital

**Keywords:** Ultrasound, Computed Tomography, Acute appendicitis

## Abstract

**Background:**

Acute appendicitis is a common cause of hospital admission and emergency laparotomy among children and young adults. Although the diagnosis is clinical, the use of radiological imaging has emerged over the past decades. Its principal use is as a problem-solving tool in equivocal cases. Owing to the increased use of imaging in the last few years, the negative appendicectomy rate has dropped significantly. In this prospective observational study, we compared the diagnostic accuracy of Ultrasonography and Non-Contrast Computed Tomography.

**Method:**

One hundred and eighteen patients with clinically suspected appendicitis followed a designed protocol. Patients underwent appendicectomy after a first performed positive ultrasonography or after a positive Non-Contrast Computed Tomography when Ultrasonography was equivocal or nonspecific. When any other diagnosis was apparent in either imaging modality which could explain the symptomatology in the patient, they were considered negative for acute appendicitis and treated accordingly.

**Results:**

The respective sensitivity, specificity, and accuracy for Ultrasonography, Non-Contrast Computed Tomography, and the whole diagnostic pathway for the diagnosis of acute appendicitis were 70.73%,80.83%, and 78.54; 100%,100%,100%, and 83.6%; and 100%,83.33% and 94.92%.

**Conclusion:**

Using Ultrasonography as the first-line diagnostic tool and Non-Contrast Computed Tomography as a complementary second-line diagnostic tool, appendicitis can be diagnosed with high accuracy and the negative laparotomy rate can be brought down significantly without any increase in the risk of complications. Computed Tomography is superior to Ultrasonography for the diagnosis of acute appendicitis.

## Introduction

The various causes of abdominal pain can range from benign to life-threatening conditions. Time is a critical factor in patients with acute abdomen, as any delay can lead to serious consequences in some cases, such as perforation, sepsis, morbidity, and mortality (1). Acute appendicitis (AA) is the most common cause of acute abdomen requiring emergency surgical intervention (2). It is usually accompanied by vomiting, fever, and diarrhea, but the most worrisome symptom is the pain. In women of reproductive age group, the diagnosis is difficult because gynecological problems can cause AA-like abdominal pain, making it a real challenge to exclude rather than to diagnose a positive case (3). Due to the serious complications, early diagnosis and intervention in AA are essential. Research is underway to develop more accurate and reliable methods of diagnosing appendicitis. The results of Computed Tomography (CT) are promising, but there are concerns about the suitability of this method in children and women of childbearing age due to radiation hazards. Although some comparative studies are present comparing the utility of Ultrasonography (USG) and CT, only a few studies have addressed a structured diagnostic algorithm (4). Even rarer are studies using non-contrast CT. The diagnostic algorithm we used was to compare the combined approach with the individual approaches and their impact on the clinical management of patients. The goal is to achieve good diagnostic results with minimal risk to patients. Our study tried to analyze the role of USG and CT in reducing unnecessary surgeries. It can be used as a starting point for larger-scale studies regarding the issue of negative appendicectomies.

## Materials and Methods

This was a prospective, hospital-based, observational study conducted at North Bengal Medical College and Hospital, West Bengal, India, from January 2021 to January 2023, after institutional ethical committee approval. The inclusion criteria considered all patients with a clinical diagnosis of AA. The exclusion criteria included patients who required emergency surgery without time for imaging, patients who previously had an appendectomy, patients who were pregnant, patients with known other bowel or pelvic masses, and patients with any proven complication of AA on imaging like perforation, abscess, or phlegmon. Patients with conservative treatment without surgery after a diagnosis of AA were excluded. Patients were first subjected to USG. Ultrasound equipment (LOGIQ P9, GE Healthcare, Cambridge, UK, Manufacturing date - 2018) with a low-frequency convex array probe (2-5 MHz) and a high-frequency linear array probe (6-15 MHz) was used for scanning. USG diagnosis of AA was based on the criteria of Jeffery et al (5) which included: a non-compressible, immobile, blind-ended tubular structure with a targetoid appearance in transverse view having a diameter more than or equal to 6 mm. Supportive features included inflamed peri appendiceal fat, collection, and appendicolith. At each USG, the following findings were noted: visualization or non-visualization of the appendix, the diameter of the appendix if visualized, compressibility, presence of appendicoliths, peristalsis, or air in the appendiceal lumen, presence of peri appendicular inflammatory changes such as an increased surrounding omental echogenicity, cecal pole edema, extraluminal fluid collection, abscess or phlegmon, extraluminal air, and lymph nodes. presence of any alternative diagnosis, which can explain the symptomatology in the patient.

Patients were classified as equivocal for AA if the appendix could not be seen but there were inflammatory alterations in the RIF and supportive evidence of AA on the USG. Nonspecific for AA patients were those in whom the appendix was visible but seemed normal, or in whom the appendix was not visible on USG but there were no supporting indications of AA on USG, and no other diagnosis could be made. Patients for whom an other diagnosis that could explain their symptoms could be proven were classified as having an alternate diagnosis. Patients with a diagnosis of AA on USG underwent appendicectomy and patients with equivocal or non-specific USG diagnosis were subjected to a Non-Contrast Computed Tomography(NCCT) abdomen.

CT was performed on a 128-slice spiral CT scanner (Revolution EVO, GE Healthcare, Cambridge, UK) at 120-KV tube voltage and 350-mA tube current. A plain CT scan was performed in the whole abdomen with a 5-mm layer thickness and a 5-mm layer distance. After scanning, multi-planar reconstruction and curved planar reconstruction were conducted at a layer thickness of 1.25 mm. All images were analyzed using a soft tissue window (window width 300-380 HU, window level 50 HU). At each CT the following findings were noted: visualization or non-visualization of the appendix, the diameter of the appendix if visualized, the position of the appendix, presence of appendicoliths in the appendiceal lumen, air in the appendiceal lumen, presence of peri appendicular inflammatory changes such as surrounding fat stranding, cecal wall thickening, extraluminal fluid collection, extraluminal air, and lymph nodes, presence of any alternative diagnosis, which can explain the symptomatology in the patient.

A CT scan was considered positive for AA in which the outer diameter of the appendix was more than 6mm. The presence of secondary signs was also noted including peri appendiceal inflammatory changes, cecal wall thickening, and appendicoliths (6). Negative on CT scan criteria for acute appendicitis was defined as the absence of signs suggestive of acute appendicitis with or without the presence of any other alternate diagnosis on CT that could explain the cause of pain in the patient. Patients with AA on CT scans too underwent appendicectomy. The patients with an alternate diagnosis in any imaging modality were considered to be negative for AA.

Histopathology (HPE) reports of all appendicectomy specimens were collected and compared with the imaging findings. Sensitivity, specificity, positive predictive value, negative predictive value, and accuracy were computed, and comparisons were made. The USG and CT were done and interpreted by the same person, a professor in radiodiagnosis with experience of over 30 years in the field.

**Ethics**: Approval was obtained from the Institutional ethical committee Ethical standards were followed as per the Declaration of Helsinki.

## Results

A total of 118 cases were clinically diagnosed with AA and referred for imaging studies. Out of the 118 patients, 64 (54 %) had an AA diagnosis based on USG. In 17 cases (14.4%), an alternative diagnosis was made. In 13 cases (11.0 %) had nonspecific findings, and 24 cases (24.3 %) had equivocal USG results. An abdominal unenhanced CT scan was performed in every case where the USG result was equivocal or nonspecific. Among 24 patients (64.9%), AA was identified at CT. Only 88 of the 118 cases underwent surgery, and the remaining patients received an alternate diagnosis. Out of the 30 cases, 11 cases were found to have a right renal calculus, 5 cases each had pelvic inflammatory disease and hemorrhagic cysts, and 2 cases each had acute pyelonephritis and acute intussusception. In these 88 cases, sonography identified AA in 64 instances, and 61 of these cases had their diagnoses supported by histopathology. Among the 37 cases who underwent CT scans 24 cases were diagnosed with AA and then confirmed by histopathology. Percentage-wise, the largest number of subjects were in the age group of 15 to 30 years (44.9%), and 1.6% were over 60 years of age. The mean age of the study population was 26.2 years with a standard deviation (SD) of 14.5. The maximum and minimum ages of the study population were 71 and 4 years, respectively. In the study population, 59.3% were males and 40.7% were females.

Males in the age group of 15 to 30 years made up the majority of AA cases. The majority of cases among females occurred in the same age group. The age group above 60 years old saw the least cases for both sexes. The most common complaint was fever, which affected all patients to some extent (100%) and was followed by backache (66.1%) and nausea (57.6%). Loose stools were the least frequently reported symptom, occurring in 19% of cases. Among the study population, 68.6% of the participants reported right iliac fossa pain, and 25.4% reported periumbilical pain. In the study population, AA was the most frequently identified USG diagnosis in 54.2% of cases, followed by equivocal findings in 21.2% and non-specific findings in 10.2% of cases, all of which required additional imaging.

The largest diameter of the appendix on USG among AA patients was 9mm and was seen in 21.8% of the population followed by 8mm in 17.1%. An additional 4.6 % had a diameter of 14mm, while 4.6 % had a diameter of less than 6mm. On USG, 68.7% of the population showed an appendicolith. In 98.4% of cases, the appendix was not compressible. The appendix was aperistaltic in all the cases of AA (100%), and in 92.1% of them, the lumen of the appendix was devoid of air. On USG, peri appendiceal inflammatory changes were present in 96.8% of cases.

The most frequent diameter of the appendix visualized on CT scans among the study population was in the range of 6 to 10 mm (51.3%) with 9mm being the single most frequent diameter (25%). In 58.3 % of cases, the appendix was found in the retrocecal position on the CT scan, while in 4.1 % of cases, the position was subhepatic. An appendicolith was present in 37% of the patients. In the study population with CT-diagnosed AA, 25% had air in the appendiceal lumen and 87.5% had peri appendiceal inflammatory changes. Among those who underwent CT 72.9% had peri appendiceal fat stranding.

AA was diagnosed by CT in 87.5% of patients with an equivocal USG finding and in 23.0% of patients with a nonspecific USG finding. Among patients with CT-diagnosed AA 91.7% had peri appendiceal fat stranding, while 8.3% did not. 38.5% of patients with a CT diagnosis, other than AA, had peri appendiceal fat stranding. Among USG-diagnosed AA patients, 90.6% had AA features on HPE. In patients diagnosed with AA by USG; those with an appendiceal diameter of less than 6 mm did not showed AA on HPE. All of the patients diagnosed with AA on CT were positive for AA on HPE. The mean appendiceal diameter on USG in patients with USG-diagnosed AA was 8.75mm with an SD of 2.12mm. The maximum and minimum diameters were 14mm and 5mm, respectively. The mean appendix diameter for all patients who underwent CT was 6.97mm with an SD of 2.86mm. The maximum and minimum diameters were 13mm and 3mm, respectively. In patients with CT-diagnosed AA, the mean appendiceal diameter was 8.5mm with an SD of 2.27mm. The maximum and minimum diameters were 13mm and 5mm, respectively. A significantly greater proportion of patients with appendiceal diameters between 6–12mm had histological evidence AA. Table 1 shows the validity of USG in the diagnosis of AA. The sensitivity, specificity, positive predictive value (PPV), and negative predictive value (NPV) of USG were 70.7%, 83.3%, 90.6% and 55.6%, respectively. The accuracy rate was 78.5%. Sensitivity, specificity, PPV, and NPV were 100% for unenhanced CT scans. Accuracy was 100%.

**Table 1:** Validity of USG in predicting AA on HPE (n=118)

Appendicitis on USG	Acute Appendicitis on HPE	
	Present	Absent
**Present**	58	6
**Absent**	24	30

Table 2 shows the validity of the combined imaging pathway for the diagnosis of AA. The sensitivity, specificity, PPV, and NPV were 100%, 83.3%, 93.2%, and 100%, respectively. The Combined path was 94.9% accurate.

**Table 2:** Comparison of the presence of appendicitis with the presence of appendicitis on any diagnostic modality (n=118)*

Appendicitis on any imaging modality	Acute Appendicitis	
	Present	Absent
**Present**	82	6
**Absent**	0	30

## Discussion

Of the 118 patients included in the sample, the majority (44.9%) were in the 15-30 years age group. B. Sulu et al, concluded that appendicitis was more common in males aged 10 to 19 years, which was consistent with the results of our study (7). Alegbeleye et al found that the overall mean age was 28.64 years with a mean SD of 10.12 years (8). The maximum percentage of the population studied was male 50.3%, again consistent with the study by B.Sulu. The male-to-female ratio was 1.4:1. Among the study population presenting with acute abdomen, AA was diagnosed in 74.5% of cases which is consistent with the study by Ademe et al(72.3%). (9)

The AA diagnosis was confirmed by USG in 54.2% of cases. Alireza et al. reported that preoperative ultrasound diagnosed 51.3% of AA cases. The appendix was seen in all patients who underwent CT, regardless of diagnosis. Keyzer et al identified it among 96.1% of their cases (10). In patients with USG-diagnosed AA, the mean USG appendicular diameter was 8.75mm, with a mean SD of 2.12mm. The maximum and minimum diameters were 14mm and 5 mm respectively. This is consistent with the studies of Shrestha et al. and Trout et al. Among the CT-diagnosed AA patients, 9mm was the most common appendiceal diameter (25.0%). This is consistent with the studies of Willekens et al. and Jayaraja et al. (11) I.G Tatar found this value to be 8.5 ± 2.7 mm (SD). (12)

Leite et al proposed that the appendiceal diameter should not be used as an absolute cutoff for AA diagnosis, as it should be interpreted in the context of clinical and other CT findings. Konodo et al found the most common position of the appendix as pelvic, in 93 cases (45.4%)(13). S. Khatun et al found the prevalence of retrocaecal appendix among patients with appendicitis was 95 (35.98%). Similarly, other positions in that study were pelvic in 67 (25.37%), post-ileal in 61 (23.10%), pre-ileal in 11 (4.16%), and subcaecal in 30 (11.36%) individuals (14). In our study, the most common position was retrocecal (58.3%) followed by pelvic (20.8%). Among patients with equivocal findings on USG, 21 out of 24 patients (87.5%) were diagnosed with AA on CT. Ramarajan et al reported 25.1% of patients with suspected AA had equivocal findings on USG and were found to have AA on CT(15). These differences in results may be due to the wide age range represented in the study (1–22 years), but only 20 patients were older than 18 years and only one patient was older than 22 years. Also, only 14 children were younger than 2 years. In addition, they had defined ambiguous USG as complete non-visualization of the appendix, which was different from the definitions we used.

Poortman et al conducted a study in 2009 on 151 patients with clinically suspected AA. Patients underwent operations after a primary performed positive USG or after complementary CT when the USG was negative or inconclusive They found 21 out of 60 patients (35%) with negative or inconclusive USG to be having AA on CT which was also proven with HPE (4). This difference may again have been due to the clubbing together of the inconclusive USG and the negative USG criteria. HPE study showed AA in 58 out of 64 cases (90.6 %) who were diagnosed with AA on USG. Shreshtha et al found USG to have a sensitivity of 85.7% and specificity of 88.2% in the diagnosis of AA with PPV of 96.4% and NPV of 62.5%(16). Inamdar et al found the accuracy and sensitivity of USG in the diagnosis of AA to be 95% and 95.83% (17). In our study, the sensitivity, specificity, PPV, NPV, and accuracy were 70.73%, 83.33%, 90.62%, 55.56%, and 78.54, respectively. Our study showed a strong correlation between appendiceal diameter on USG, and positive HPE diagnosis of AA.100% of cases with a diameter below 6mm were negative on HPE. This is in concordance with the study of Shreshtha et al and N.H. Park et al (18).

Rajeev Sharma et al found NAR to be 23.7% and suggested the use of CT (19). In our study, the NAR with USG was 9.37%, and with combined USG and CT was 6.81%. Among patients diagnosed with AA on CT, all 100% had a diagnosis of AA on HPE. Rud et al found the probability of having AA following a positive CT result and after a negative CT result to be 0.92 and 0.04, respectively (6) N.R. Singh et al found the sensitivity and specificity of NCCT in the diagnosis of AA as 98.2% and 100%, respectively (20). A systematic review by Terasawa et al concluded that CT was more accurate for the diagnosis of AA than USG with an overall sensitivity of 0.94 and overall specificity of 0.95. (21)

The most common alternative diagnoses on USG were hemorrhagic cysts (HC) and PID, with HC being seen as cysts with fishnet-like contents and PID as an endometrial collection with an adnexal mass. The most common alternate diagnosis on CT scan was renal colic due to right renal calculi. In most of these cases, the sizes of the calculi were less than 5mm. The sensitivity of USG for the detection of renal calculus less than 5mm is low as it is often confused with sinus fat. Ileocecal tuberculosis is very common in India and is seen on CT as wall thickening, thickened ileocecal valves, and fibrosis as USG findings here are highly nonspecific. The main limitation of our study was the non-availability of HPE reports in all the clinically suspected cases and a small sample size that underwent CT scan. The sensitivity and specificity of USG and CT in our study were comparable to those in other studies. CT was superior to USG in the diagnosis of AA.

USG has advantages over CT such as high soft tissue contrast, better wall pattern and wall thickness evaluation, ease of performance, ready availability, cost-effectiveness, and the potential for discovering other causes of abdominal pain. The main disadvantages of USG are operator dependency and the need for considerable expertise. CT has advantages such as high resolution, reproducibility, higher accuracy, and better identification of both the normal and inflamed appendix and its complications like phlegmon and abscesses. The disadvantage of CT is radiation hazard and non-availability in all places, unlike USG.

We recommend using USG as the first-line imaging investigative modality in all cases of suspected AA and CT to be used in equivocal or negative cases. This is after considering the risk of negative appendicectomy and increased radiation exposure against the complications of delayed diagnosis and subsequent morbidity. The risk of complications associated with delayed diagnosis largely outweighs the small risk of radiation hazard.

## Figures and Tables

**Figure 1:**
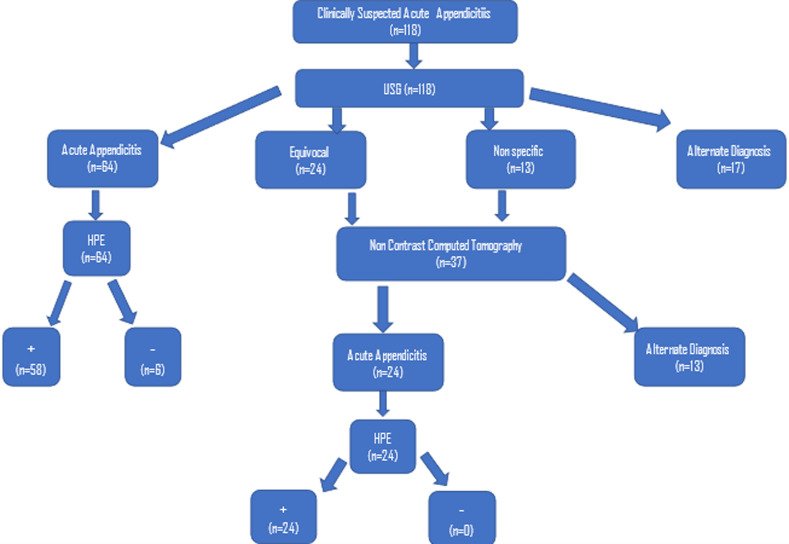
Study protocol with the number of patients recruited at each step

**Figure 2:**
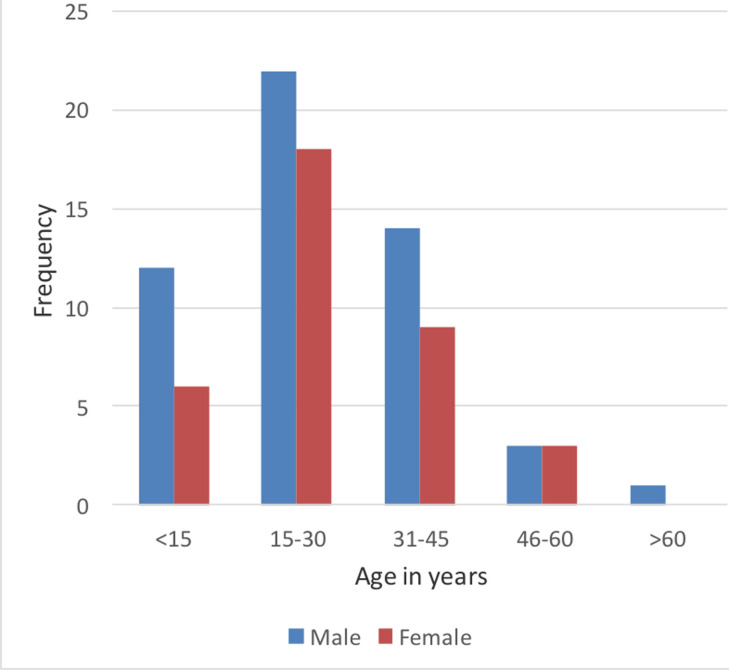
Age and Sex distribution of the study population
